# Directional Migration and Distribution of Magnetic Microparticles in Polypropylene-Matrix Magnetic Composites Molded by an Injection Molding Assisted by External Magnetic Field

**DOI:** 10.3390/ma15134632

**Published:** 2022-07-01

**Authors:** Hang Gu, Guofeng Qin, Anfu Chen, Mingke Li, Dejie Huang, Zhangyuan Peng, Jingjing Zhang, Caihong Lei

**Affiliations:** Guangdong Provincial Key Laboratory of Functional Soft Condensed Matter, School of Materials and Energy, Guangdong University of Technology, Guangzhou 510006, China; 3119005925@mail2.gdut.edu.cn (H.G.); kun19980909@163.com (G.Q.); jy9849yz@163.com (M.L.); 3120005734@mail2.gdut.edu.cn (D.H.); 3119005938@mail2.gdut.edu.cn (Z.P.); zhangjj@gdut.edu.cn (J.Z.); lch528@gdut.edu.cn (C.L.)

**Keywords:** directional migration, magnetic microparticles, polymer composites, injection molding, external magnetic field

## Abstract

Surface-functionalized polymer composites with spherical particles as fillers offer great qualities and have been widely employed in applications of sensors, pharmaceutical industries, anti-icing, and flexible electromagnetic interference shielding. The directional migration and dispersion theory of magnetic microparticles in polypropylene (PP)-matrix magnetic composites must be studied to better acquire the functional surface with remarkable features. In this work, a novel simulation model based on multi-physical field coupling was suggested to analyze the directed migration and distribution of magnetic ferroferric oxide (Fe_3_O_4_) particles in injection molding assisted by an external magnetic field using *COMSOL Multiphysics*^®^ software. To accurately introduce rheological phenomena of polymer melt into the simulation model, the Carreau model was used. Particle size, magnetic field intensity, melt viscosity, and other parameters impacting particle directional motion were discussed in depth. The directional distribution of particles in the simulation model was properly assessed and confirmed by experiment results. This model provides theoretical support for the control, optimization, and investigation of the injection-molding process control of surface-functionalized polymer composites.

## 1. Introduction

The properties of polymer matrix composites consisting of the pure polymer and functional fillers can be adjusted via varying the content and distribution of the fillers [1,2,3,4]. Generally, micro- and/or nano-sizes of organic, inorganic, and metallic granular fillers are added into a pure polymer as functional reinforcement fillers for the fabrication of polymer composites [5]. The addition of thermally conductive fillers and magnetic fillers enables polymer composites to gain conspicuous thermal conductivity and magnetostrictive properties, respectively [6,7]. These polymer composites can be widely used in the fields of sensors [8], microelectronics packaging materials [9], pharmaceutical industries, flexible electromagnetic interference shielding materials, and other fields [10,11,12]. Bian et al. [13] prepared an epoxy resin composite with 22.5 wt.% boron nitride and 7.5 wt.% nano Al_2_O_3_ particles, which resulted in a seven-fold increase in thermal conductivity and a four-fold increase in breakdown time compared with the neat epoxy. This suggests that the outstanding composite could be used as an insulating material in the electronics and electrical industries. In one syllable, it has been demonstrated that the addition of various functional particles enhances the various composites’ properties to a certain extent in numerous scholars’ research. This mainly occurs in thermal [14] and electromagnetic interference shielding [15]. The mechanical properties generally increased in impact resistance and strength, while tensile and bending properties decreased [16,17]. Generally, the directional arrangement of the granular phase in the polymer further contributes to the enhancement of the combination properties in thermal, electrical, and mechanical with outstanding anisotropy [18,19,20,21,22]. The excellent properties of these polymer composites have attracted extensive attention. As ideal manipulation methods of the directional distribution of the granular phase, using external field-assisted molding technologies such as magnetic fields or electric fields [23], some surface-functionalized polymer composites with specific properties can be obtained.

Taking advantage of the different physical properties of the polymer matrix and functional fillers, the external field is likely to strengthen the functional behaviors of the polymer composites. Volpe et al. [24] used a magnetic field to induce a magneto-sensitive part made of a thermoplastic elastomer reinforced with iron microparticles. Chen et al. [25] applied a magnetic field to pull magnetic photothermal particles towards the surface to shorten the photothermal transport path and increase the surface temperature rise effect. These external magnetic field-regulated polymer composites have excellent application in the fields of anti-icing, microwave absorption [26], and self-cleaning [27]. As an ideal method widely used in high-efficient, low-cost, and rapid large-scale production, injection molding promoted the application of polymer composites to be further expanded and burgeoned [28,29]. The abovementioned researches have mainly paid attention to the preparation, performance, and application of polymer composite materials. However, to obtain polymer composites with better surface functionalization, the mechanism for the interaction force and migration of particles in the external field-assisted molding process should be clarified, especially for the polymer composites molded via injection molding.

The finite element method (FEM) is a widely used numerical technique for solving engineering and physics problems involving behaviors that can be described by differential equations [30]. Because granular fillers were scattered and tiny, finite element analysis software was usually used to simulate the motion of granular fillers. Applying the finite element multi-physical field simulation software *COMSOL Multiphysics*^®^, the trajectory of moving particles can be visualized distinctly, making it possible to model the realistic filling process of injection molding, accompanied by better understanding and optimizing processes for the real situation. Sharma et al. [31] developed a mathematical model and used *COMSOL* to model and then trace the magnetic particles during the transport of magnetic drugs through blood vessels. Wang et al. [32] used a fully coupled multi-physics model to study the dynamic behavior of various magnetic particles in magnetic separators. Most of the fluids used in the previous research studies are Newtonian fluids with constant low viscosity, such as blood, water, and so on, but this previous work has great realistic significance and guiding role in the trajectory of moving particles for this paper. Additionally, the conventional mesh elements for the flow field at the micro-scale were certainly not refined to match the micro-scale magnetic particles. This may reduce the accuracy of the results of the simulation. Consequently, the varying high viscosity of the polymer melt with shear-thinning behavior during the injection molding is a challenge for the trajectory of moving particles [33]. So far, there have been few relevant reports on the modeling motion of magnetic particles in the high viscous polymer during injection molding. In the present work, a viscous fluid consisting of a high viscosity polypropylene (PP) melt and a certain mass fraction of magnetic microparticles was developed, and it was filled into the cavity in the tool under the external magnetic field-assisted injection molding. The local mesh refinement method was used to match the mesh size with the particle size, resulting in a slightly increased calculation amount but significantly improved the precision of the result. Directed migration and aggregation of magnetic particles were performed, and the trajectory of moving particles was visualized and analyzed by using the *COMSOL Multiphysics*^®^ software. Moreover, the experimental results fit with the simulation results, and so the accuracy of the simulation model was verified. Besides, the metastable behavior of particles, which cannot be obtained experimentally, was investigated via a modeling parametric process. Through the simulation software, the force analysis and pursuit tracking of microparticles were established during the molding process, aiming to help guide the molding process and provide theoretical support for the fabrication of these kinds of magneto-sensitive polymer composites.

## 2. Materials and Methods

### 2.1. Materials

Magnetic ferroferric oxide (Fe_3_O_4_) powders (average diameter: 50~300 nm, 97% trace metals basis) were purchased from Shanghai Aladdin Biochemical Technology Co., Shanghai, China. The polymer used as received in this work was commercial PP (grade CJS700, Sinopec Corp. Guangzhou, China). It has a melt flow index (MFI) of 11.0 g/10 min (230 °C, 2.16 kg).

### 2.2. Preparation of PP/Fe_3_O_4_ Composites

**Figure 1:**
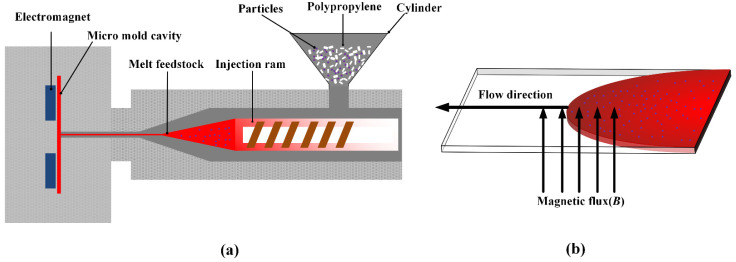
(**a**) Schematics of injection molding process under an external magnetic field, (**b**) schematics of magnetic field direction and melt filling direction, the ***B*** is the magnetic flux density.

The PP/Fe_3_O**_4_** composites with an Fe_3_O**_4_** content of 5 wt.% were prepared via injection molding under the external magnetic field. Figure 1 shows the schematics of the mixing and injection molding process of the composites and the magnetic flux applied in the microcavity during the process. The melt temperature and mold temperature were set at 210 °C and 60 °C, respectively. A mold equipped with a temperature control apparatus and an electromagnet control was used to mold a rectangular substrate with nominal dimensions of 400 × 150 × 10 mm^3^. The electromagnet was turned on and the temperature was set at 180 °C before the melt-injection. After the mold was closed, the melts were filled. Finally, the magnetic attraction did not disappear until the melt cooled down for 2 min. Experiments were conducted at a mold temperature of 230 °C.

### 2.3. Characterization

The surface morphology of PP/Fe_3_O_4_ composite samples was characterized by a field-emission scanning electron microscope (FE-SEM, Hitachi SU8220, Tokyo, Japan) at an accelerated voltage of 15 kV. Before SEM observations, samples were coated with gold by ion sputtering. The distribution of Fe_3_O_4_ particles was further characterized by an energy-dispersive spectrometer (EDS).

## 3. Numerical Model

### 3.1. Magnetic Fields

The finite element software analysis method is the most comprehensive and effective approach to the mathematical and engineered model solutions at microscale. A disc-shaped permanent magnet (N50, Sintered NdFeB) was used as the electromagnetic field. Because the particle itself has little charge, it is feasible to use the “Magnetic Fields, No Currents (MFNC) module” interface to simulate the magnetophoretic force of particles. It was a great challenge to characterize the filling behavior of polymer melt during injection molding since the rheological behavior of non-Newtonian fluids at high shear rates as well as the microchannel was hard to describe by a specified formula. In this case, the Carreau viscosity model was used to characterize the rheological behavior of the PP/Fe_3_O_4_ melt.

In a current free region, analogous to the definition of the electric potential for static electric fields, it is possible to define the scalar magnetic potential, *V***_m_**.

(1) ∇×H=0

(2) $$H=-\nabla V_{m}$$

where ***H*** is the magnetic field intensity, the constitutive relationship between magnetic flux density, magnetic field, and remanent flux density was used in Equation (3) as follows:

(3) $$B=\mu_{0}\mu_{\mathrm{rec}}H+B_{r}$$

where the *μ*_0_ is the magnetic permeability of vacuum, the *μ*_rec_ is the recoil permeability determined by the intrinsic property of the electromagnet material (N50) then ***B*** is the magnetic flux density, and ***B***_r_ is the remanent flux density norm outside and inside the magnetic region varying from 0 to 0.73 T. According to the Equations (1)–(4), we can derive the following Equation (5):

(4) ∇⋅B=0

(5) $$\nabla \cdot (\mu_{0}\mu_{\mathrm{rec}}\nabla V_{m}-B_{r})=0$$

The equation mentioned above describes the magnetic field generated by a permanent magnet. To limit the region of magnetic fields, establishing an adequate permanent magnet magnetic field model necessitates the proper boundary conditions. Therefore, reasonable boundary requirements must be considered. The magnetic insulation condition, which sets the normal component of the magnetic flux density to zero, can be used to explain the boundary conditions as follows:

(6) $$n\cdot (\mu_{0}\mu_{\mathrm{rec}}\nabla V_{m}-B_{r})=0$$

### 3.2. Melt Flow in the Cavity

Creeping Flow, also referred to as Stokes flow, occurs in melts with high viscosity or small geometrical length scales. The “Creeping Flow” interface was used to simulate fluid flows at very low Reynolds numbers where the inertial term in the Navier−Stokes equations can be neglected. In this work, the “Creeping Flow” interface in *COMSOL* was used. It can well represent the dynamic characteristics of inelastic and non-Newtonian fluids during injection molding. For this type of flow, the Carreau viscosity model and the related parameters were used and are listed in Table 1. The equations solved by the “Creeping Flow” interface are the Stokes equations for conservation of momentum and the continuity equation for conservation of mass as follows:

(7) $$0=\nabla \cdot \left[-pI+\mu_{\mathrm{ap}}(\nabla u+{\left(\nabla u\right)}^{T}\right]+F$$

(8) ρ∇⋅u=0

where *μ*_ap_ is the apparent viscosity, *p* is the pressure, *u* is the local velocity, *T* is the melt temperature, ***I*** is the stress tensor, ***F*** is the volume force, and *ρ* is the density of the fluid. For Carreau fluid, the constitutive relation between viscous stress *K* and strain−tensor *S* was given by Equation (9) as follows:

(9) $$K=\left(\mu_{\infty }+(\mu_{z}-\mu_{\infty }){\left[1+{\left(\lambda \gamma \right)}^{2}\right]}^{\frac{\left(n-1\right)}{2}}\right)S$$

where *γ* is the shear rate, *μ*_∞_ is the infinite shear rate viscosity, *μ*_z_ is the zero shear rate viscosity, *λ* is the relaxation time, and *n* is a dimensionless parameter. The strain-tensor *S* is related to the following two equations:

(10) $$\dot{\gamma }=\sqrt{2S:S}$$

(11) $$S=\frac{1}{2}\left[\nabla u+{\left(\nabla u\right)}^{T}\right]$$

Usually, the normal direction of the inlet boundary is chosen as the inflow velocity direction of the fluid, and the non-slip boundary is selected on the wall (*u* = 0). The pressure is specified at the outlet so that the appropriate flow conditions can be selected for the Navier−Stokes equation and the result can be converged as follows:

(12) $$u=U_{0}n$$

(13) $$\left[-pI+\mu_{\mathrm{ap}}(\nabla u+{\left(\nabla u\right)}^{T})\right]n=-p_{0}n$$

where *U*_0_ is the normal inflow velocity, *p*_0_ is the relative pressure at the boundary, and ***n*** is the inlet boundary normal vector pointing out of the domain. The pressure condition was set to suppress backflow.

### 3.3. Tracing of Particles

In this model, only three types of forces, including drag force, magnetophoretic force, and gravity force, were taken into consideration. The physical property of particles used in this work was set to be iron powder from the *COMSOL* “Material Library” to obtain good magnetism and high relative permeability, the trajectories equation of particles was defined as follows:

(14) $$\frac{dq}{dt}=u+\frac{\tau_{p}}{m_{p}}F_{\mathrm{ext}}$$

where *q* is the particle position, ***u*** is the fluid velocity, ***F***_ext_ is the sum of applied forces other than the drag force, and *τ*_p_ is the response time of particle velocity as a measure of the time scale of the particle velocity approaching the velocity of the surrounding fluid; when particles are tiny enough and move slowly relative to the surrounding fluid, the drag force ***F***_D_ induced by the high viscosity of the fluid should be considered, and the Stokes law of drag definition can be applied as follows:

(15) $$F_{D}=\frac{1}{\tau_{p}}m_{p}(u-v)$$

(16) $$\tau_{p}=\frac{\rho_{p}d_{p}{}^{2}}{18\mu_{\mathrm{spf}}}$$

where *μ*_spf_ is the dynamic viscosity of the PP viscous fluid, the ***v***, *ρ*_p_, *d*_p_, and *m*_p_ are the velocity, density, diameter, and mass of particles, respectively. The magnetophoretic force acting on a spherical particle can be calculated by Equation (17) as follows:

(17) $$F_{M}=\frac{1}{4}\pi d_{p}{}^{3}\mu_{0}\mu_{\mathrm{pp}}K\nabla H^{2}$$

where *μ*_pp_ is the relative permeability of the magnetic fluids. The *K* is the Clausius−Mossotti (CM) factor, which uses magnetic permeability and is defined as follows:

(18) $$K=\frac{\mu_{r,p}-\mu_{\mathrm{pp}}}{\mu_{r,p}+2\mu_{\mathrm{pp}}}$$

where *μ*_r,p_ is the relative permeability of the Fe_3_O_4_ particles. Although the gravitational effect of particles in this model is small, it still cannot be ignored due to the large particle size and density. Since the density of the surrounding fluid is considered, the gravity node equation also includes buoyancy. The gravity note equation of particles in the fluid field can be expressed as follows:

(19) $$F_{G}=m_{p}g\frac{\rho_{p}-\rho }{\rho_{p}}$$

where *ρ* is the density of the fluid, and g is the gravitational acceleration. In the equation described above, the total force acting on the particle can be obtained as follows:

(20) $$F_{T}=F_{D}+F_{M}+F_{G}$$

### 3.4. Mesh and Parameters

**Figure 2:**
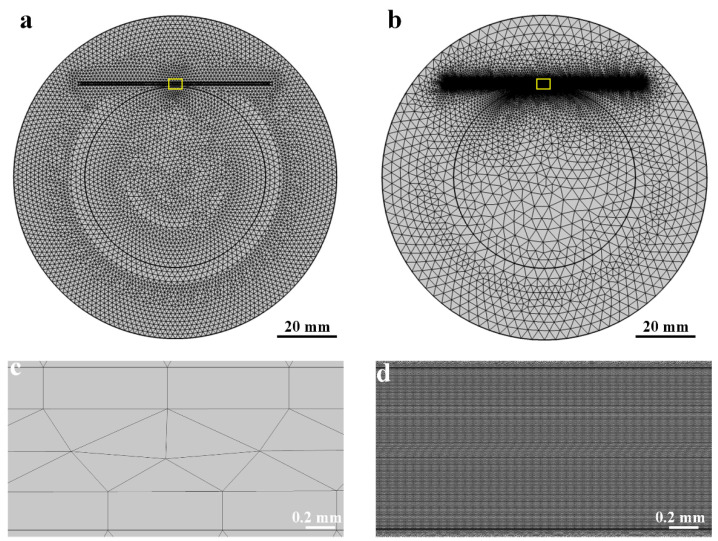
(**a**) Extremely fine mesh, (**b**) Local refinement mesh, and their corresponding enlarged (**c**,**d**) images of the mesh in the cavity (the yellow box), respectively.

Generally, the accuracy obtained based on any finite element analysis model is directly related to the finite element mesh used. Elements become smaller through the continuous refinement of the mesh so that the solution results are closer to reality. Especially for the coupling of multiple physical fields, the mesh for common physical fields or even extremely fine mesh cannot meet the computing requirements, so the selection of an appropriate mesh size by local thinning or adaptive thinning of physical fields has a prominent influence on the simulation results. Local mesh refinement can conserve computational resources while increasing the computational accuracy of crucial observation areas. In this paper, we adopted the approach of local mesh refinement to optimize the quality of the mesh, and we compared it to the basic physics-controlled mesh in terms of mesh number, element quality, skewness, and so on, with the comparative results displayed in Figure 2. Fortunately, the model was symmetric in structure. To reduce the calculation amount of the simulation analysis model, the two-dimensional section was used to represent the side diagram of the molding, and modeling analysis in this form can better characterize the migration effect of particles.

**Figure 3:**
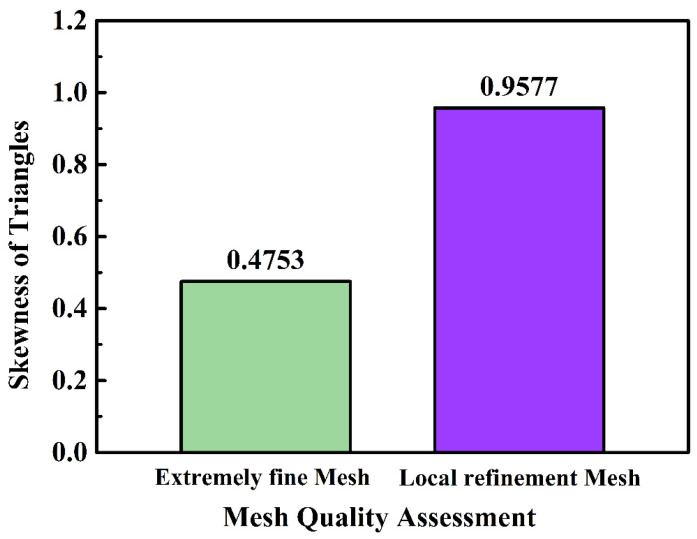
Comparison between extremely fine mesh and local refinement mesh.

The free triangular mesh is mostly employed in the injection mold cavity, whereas the boundary layer quad mesh is used on the boundary wall to account for fluid shear thinning. After the mesh local refinement, the cavity mesh had a total of 2,372,146 elements, including 2,327,162 triangles and 44,984 quads among them. This was a significant figure for such a micro-scale model. By comparison, the total number of elements in the model is only 2,682,888. The mesh quality was usually assessed by the skewness of triangles, which is a measure of the equiangular skew, and the minimum element value in the skewness (value from 0 to 1) statistics was above 0.01 as a rule of thumb. In this mesh, the average element quality of the entire model was 0.9467. This indicates that the overall simulation model, particularly considering particle size and fluid dynamics, has high computational accuracy. Figure 3 shows the comparison of the mesh quality between extremely fine mesh and local refinement mesh. The results show that the effect of mesh refinement was significant. The cavity’s mesh area was just 0.76% of the whole mesh area, yet the cavity’s elements accounted for 88.42% of the total mesh numbers after local refinement, compared to only 3.35% for extremely fine mesh. The mesh quality as measured by skewness has improved significantly as a result of mesh refinement, particularly the triangular mesh element quality, which has increased from 0.4753 to 0.9577, implying that the finite element calculation results are more convergent and the simulation model results are closer to the true value. After establishing the mesh, the *COMSOL* software’s time-dependent solver was used to study the interface. In the fully coupled model, the nonlinear approach was set constant (Newton) with a 0.9 damping factor, and the termination strategy was set to tolerance with a 0.1 tolerance factor. The rest of the model’s parameters are listed in Table 2.

## 4. Results and Discussion

### 4.1. Magnetic Field Strength and Particle Trajectories

**Figure 4:**
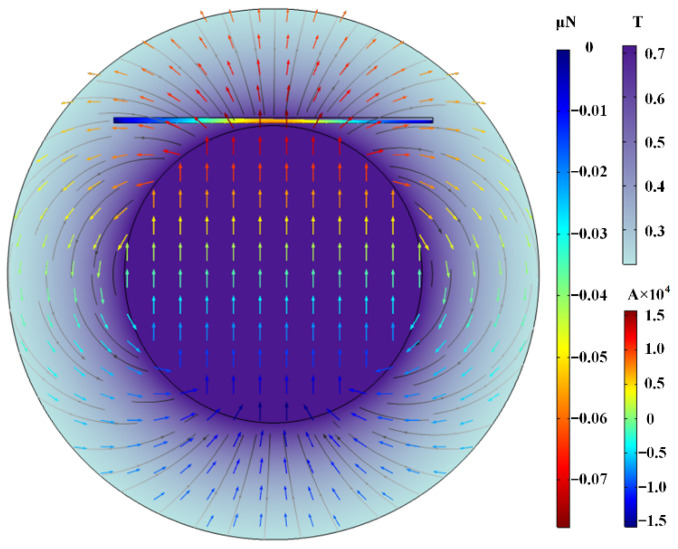
Entire 2D simulation model.

Figure 4 shows the entire 2D simulation model under the external magnetic field and the magnetophoretic force of particles. The innermost circle represents a disc-shaped magnet, and the ring belt between the inner circle and the outer circle was set as an air domain. The dimension of the cavity is 60 × 1 mm^2^, and the distance between the cavity and the magnet is 1 mm. The direction and magnitude of the magnetic field and magnetic scalar potential can be marked by black magnetic lines and colored arrows, respectively. The magnetic field distribution of the permanent magnet in space is clearly observed. The closer to the surface of the magnet, the denser the black magnetic lines are, and the greater the intensity of the magnetic field is. The magnetic scalar potential described the amount of auxiliary magnetic properties, and the direction of the arrow was consistent with the direction of the magnetic induction line. The direction was from the N pole to the S pole outside the magnet, and vice versa inside the magnet.

**Figure 5:**
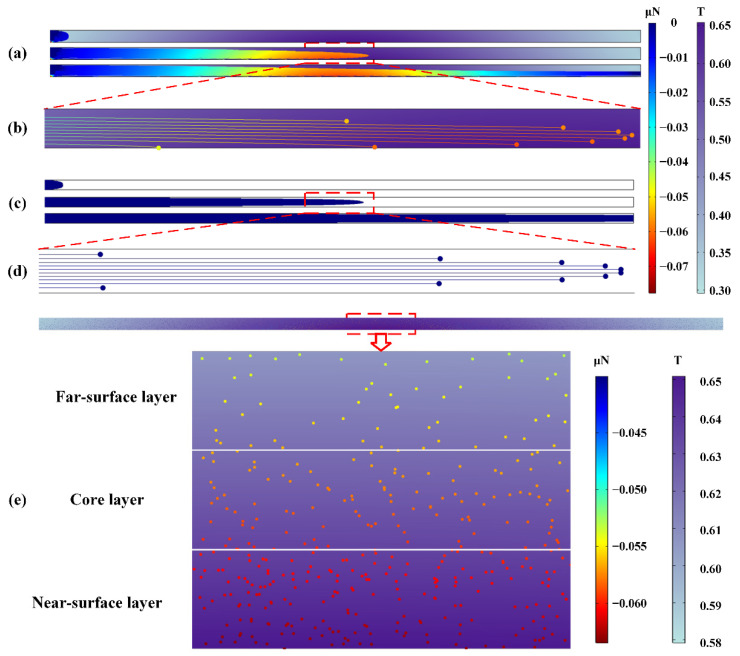
(**a**−**d**) refers to occurrences in micro mold cavity. Contrast diagrams of (**a**,**c**) injection molding process, (**b**,**d**) particle migration with external magnetic field and without magnetic field, and (**e**) particles final distribution.

Figure 5 shows the contrast diagram of the injection molding process, particle migration, and particle distribution with and without a magnetic field. The distribution of the magnetic field was not uniform. It can be observed that the intensity of the magnetic field was up to 0.65 T in the cavity center and only 0.30 T at the far end of the field. Figure 5a,c show typical images of injection molding processes with and without magnetic fields, respectively. The whole injection molding processes were recorded and are displayed in Supplementary Videos S1 and S2. Due to the high viscosity of the melt, there was a drag force as an interacting force between particles and melts. Under low-level flow index at the wall and the injection pressure gradient, the melts were filling as a fountain flow. Under the influence of the external magnetic field, the melt encasing magnetic particles migrated toward the magnetic surface. We examined particle migration in the same horizontal direction under the operation of a non-uniform magnetic field and non-magnetic to study the filling path of particles explicitly, and the results are shown in Figure 5b,d. The particles in the legend have been magnified 5~60 times their original size to make the results easier to observe. The gradient color represents the magnitude of the magnetophoretic force of the particle. The negative sign of the value indicated that the magnetophoretic force acting on the particle was a magnetic attraction force, and the variation of the magnetophoretic force on particles was highly consistent with the variation of magnetic field intensity. The trajectories of particles wrapped in the high viscosity melt were highly correlated with the filling process of the melt. Comparing with the horizontal migration of particles without magnetic field, particles moved in parabolic trajectories under the action of the magnetic field, and particles migrated toward the near-surface of the sample. Meanwhile, it can be observed that the particles near the wall moved more slowly than the particles in the core layer, which was related to the lower flow index of the melt on the wall caused by the lower temperature. Ultimately, the final distribution of particles with a magnetic field is shown in Figure 5e. The gradient color represents the magnitude of the magnetophoretic force of the particle at different positions. For better quantification and expression, we divided the sample into the following three regions from thickness direction: near-surface layer, core layer, and far-surface layer. It can be observed that the particles with magnetic field tended to scatter over the near-surface of magnetic field, and the number of particles in the near-surface region was much greater than that in other regions. The closer the particles move to the surface of the magnet, the greater the force they experience in a parabolic trajectory; then, the particles will migrate quicker toward the surface of magnet, finally, the number of particles in the near-surface region will increase.

### 4.2. Viscous Fluid Model

**Figure 6:**
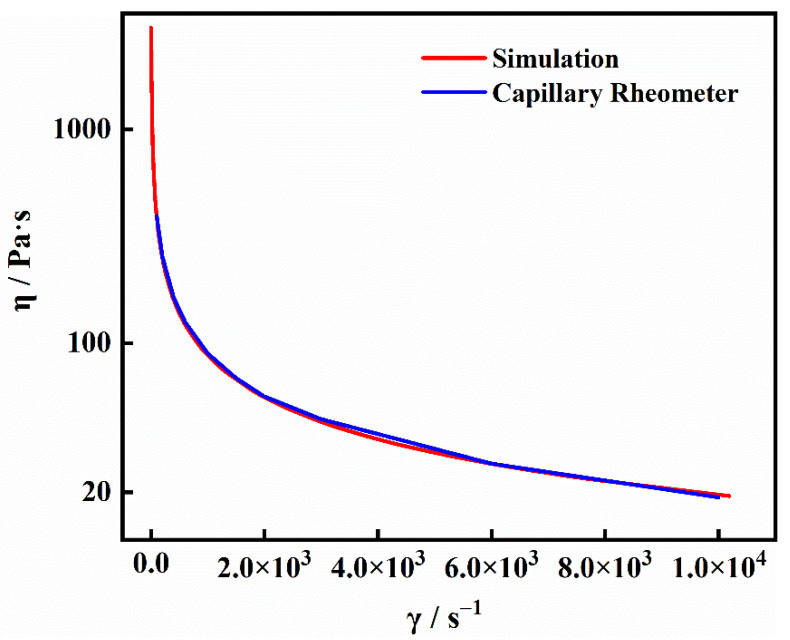
Simulated and experimental relationship relationships between shear viscosity and shear rate of PP/Fe_3_O_4_ composites with Fe_3_O_4_ content of 5 wt.% at temperature of 210 °C.

The complex rheological behavior of polymers during the injection molding process has always been the key point of relevant research. Figure 6 shows the simulated and experimental relationships between shear viscosity and a shear rate of PP/Fe_3_O_4_ composites with an Fe_3_O_4_ content of 5 wt.% at a temperature of 210 °C. The corresponding parameters can be calculated by Equation (9) and introduced into the *COMSOL* simulation model. As can be seen, these two curves coincide with each other. It indicated that the data measured by the capillary rheometer was in line with that obtained from the simulation model. Furthermore, the simulation model’s validity was proved by its good agreement with empirically established melt flow parameters, and the fluid was successfully integrated into the simulation model. Most importantly, the data range of the curves obtained by the simulation model was much wider than that of the experimental data, and the curves of the simulation data were smoother, which played an extremely important role in investigating the complex rheological behavior of composite melt during injection molding, especially in the case of extremely low shear rate and extremely high shear rates that were usually difficult to determine by using capillary rheometer [34,35]. The wider range of shear rate means that the polymer melt’s flow characteristics were extremely complex in the whole process of injection molding. For example, the shear rate of the melt at the front of the injection nozzle was eminently high, which corresponded to the low viscosity, meaning that the melt has good fluidity. Nevertheless, in the process of cooling and packing, the shear rate in the overall cavity was eminently low, corresponding to the high viscosity. This meant that the melt was cooling down qualitatively at this time, and the fluidity was very poor. At the same time, the application of the simulation model can greatly reduce the workload of filling quality evaluation during the injection molding process, compared with the method of deploying pressure and temperature sensors in the mold cavity for detecting the flow behavior of melt [36]. Because the sample’s thickness was only 1 mm, the average shear rate of the melt in the cavity was 1 × 10^4^ s^−1^ under extremely high injection pressure during injection molding. To simplify the force acting on particles during injection molding, the viscosity in the fluid drag force of particles was regarded as a constant value, which was 21 Pa·s when corresponding to the shear rate is 1 × 10^4^ s^−1^.

### 4.3. Factors Affecting Motion of Particles

According to Equations (15), (17), (19) and (20), we can easily find the factors that will affect the motion of particles most. Because the particle size was extremely small, the effect of gravity with a value as low as 5 × 10^−16^ N can be ignored compared with the other two forces.

**Figure 7:**
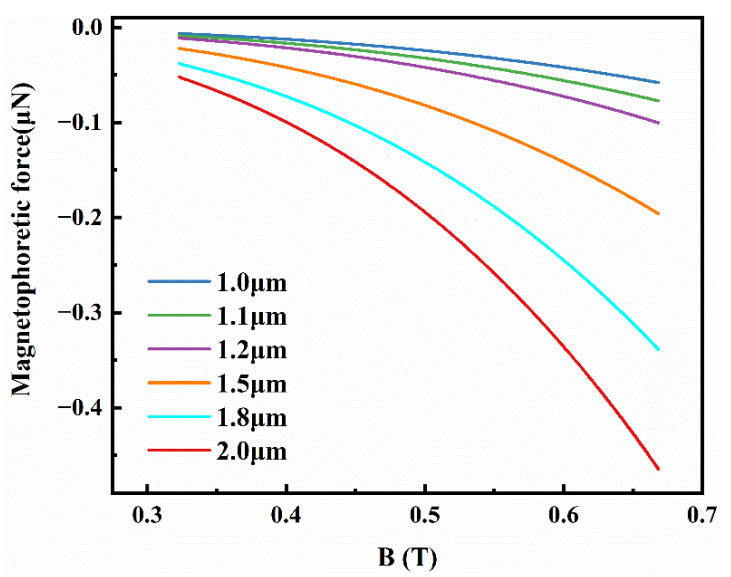
Magnetophoretic force of different particle sizes acting on diverse magnetic field intensity.

According to Equation (17), particle size, magnetic field intensity, and relative magnetic permeability are the main factors affecting particle migration to the near-surface of the sample. Figure 7 shows the relationship for different particle sizes between the magnetophoretic force of particles and magnetic field intensity. Apparently, the magnetophoretic force of particles is increased with particle size and magnetic field intensity. The particle size increases from 1 to 2 μm, resulting in an eight-fold rise in the magnetophoretic force. Similarly, the magnetic field intensity increases from 0.35 to 0.65 T, resulting in a 6.4-fold increase in magnetophoretic force. Though the relative magnetic permeability of particles was important, through using physical/chemical modification or introducing more magnetic fillers to increase the relative magnetic permeability of particles will significantly increase the overall workload too much [37,38], resulting in a substantial increase in material cost. In addition, the modified particles were likely to weaken the performance of surface-functionalized composites more or less; therefore, the influence of particle relative magnetic permeability will not be studied in this paper.

**Figure 8:**
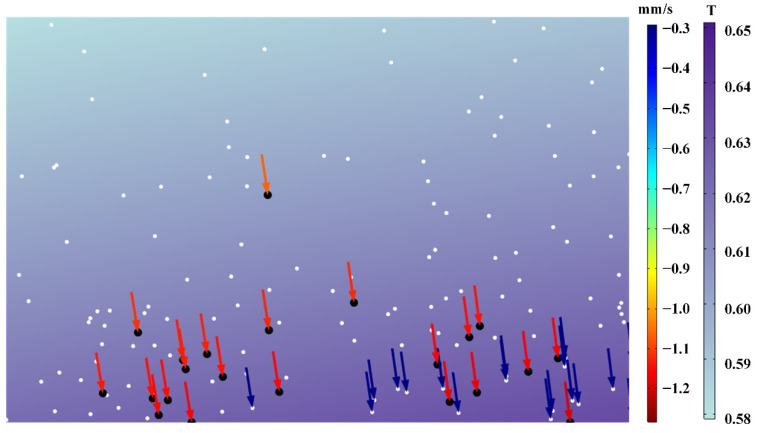
Distribution of particles with different sizes.

Figure 8 depicts a schematic diagram of the simulative distribution of magnetic Fe_3_O_4_ particles in the cavity with particle diameters of 1 and 1.5 μm, denoted by white and black circles, respectively. The color of the arrow indicates the magnetophoretic force of the particle when it was deposited under the influence of an external magnetic field. The closer the particle is to the magnet, the magnetophoretic force is greater as well as the particle velocity. The arrow direction indicates the direction of the magnetophoretic force of the particle, from small to large magnetic fields. As can be seen, the simulative distribution is close to the distribution in the experimental results, so it was used to simulate the specific experimental process. In our experiment, since Fe_3_O_4_ particles were easily magnetized and magnetic, they tended to agglomerate and form a large particle size agglomerated magnetic particle during melting. The aggregation can be approximately regarded as a larger-sized particle, which receives a larger magnetophoretic force in the magnetic field. Thus, they were more likely to migrate under the influence of a magnetic field. The larger the magnetophoretic force of particles is, the acceleration and speed of particles are also greater. This meant that the particle’s response was more rapid and sensitive under the action of a magnetic field, considering the high-speed melt filling rate in the process of injection molding. The whole sample molding cycle was in short order. Superior response and migration of particles have a crucial influence on the final distribution, as shown in Figure 8. Large-size particles tended to assemble on the surface of the sample, while small-size particles tended to stagnate due to poor fluidity caused by melt cooling.

### 4.4. Particle Tracking and Distribution

**Figure 9:**
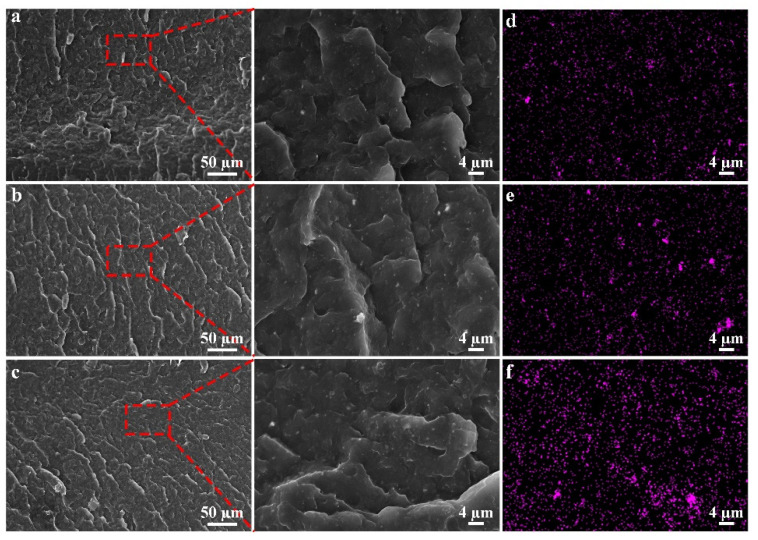
SEM images of (**a**) far-surface layer, (**b**) core layer, and (**c**) near-surface layer of specimen in cross section direction; EDS images of partial enlarged drawing of them (**d**–**f**), Purple dots represent Ferrum.

To obtain better near-surface functionalized PP/Fe_3_O_4_ composites, we focus on studying the transmission probability of particles in each regional segment. In the vertical direction, it was divided into the near-surface layer, core layer, and far-surface layer. The actual particle distribution is shown in Figure 9.

**Figure 10:**
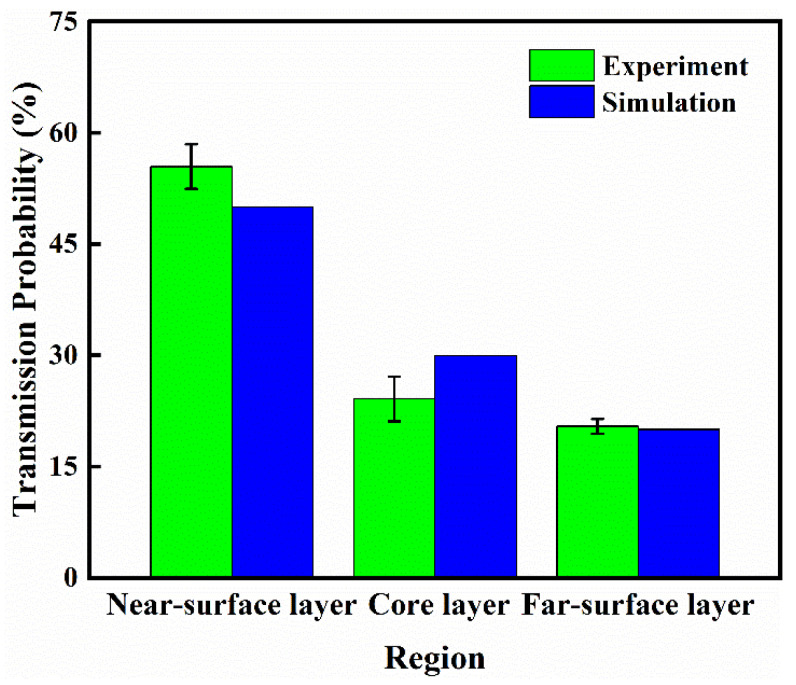
Particle transmission probability of experiment and simulation model.

Figure 9 shows that under the action of a magnetic field, magnetic Fe_3_O_4_ particles moved closer to the surface of the magnetic field and had a significant migration effect. As can be seen, white bright dots are magnetic Fe_3_O_4_ particles, and the average particle diameter is 1 μm. From the far-surface layer to the core layer and near-surface layer. The particle numbers escalate to varying degrees. Simultaneously, the closer to the near-surface layer, the more particles existed. The experimental results of the particle distribution trend were in line with the above simulation theory research. The EDS photos also confirmed this result, the number of purple dots representing ferrum increased as it got closer to the near-surface layer, and partially purple dots with larger areas representing particle aggregation were also tending to accumulate in the near-surface layer. The actual distribution of particles in each layer can be estimated and calculated by quantifying the number of particles in the area of purple dots measured by Image-Pro software. Through simulation, the number of particles on each layer can be counted and compared with the experiment result. The result of the transmission probability of the experiment and simulation is displayed in Figure 10. The fact that simulation results matched well with experimental results verified the accuracy of the simulation model and provided theoretical support for the subsequent use of the simulation model to assist in improving the actual molding process. On the particle distribution in the far-surface layer, there was a high degree of consistency between simulation and experimental results, although there was a 5% deviation between core-layer and near-surface layer data. The simulation mold was set up in the center of the cavity in the normal direction, and the injection fluid was “fountain flow,” thus the velocity of the fluid in the middle was much higher than the fluid nearing both sides’ walls. Particles in the magnetic field had a shorter action time as a result of this, and particles in the core layer had a shorter migration distance. Finally, the core layer had greater amounts of particles. As a result, the number of particles in the core layer was marginally reduced in the simulation results compared to that in the experiment.

### 4.5. Metastable Analysis

**Figure 11:**
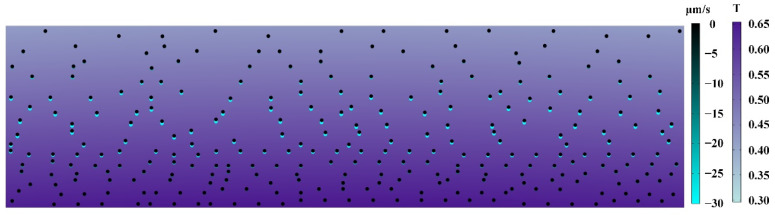
Metastable motion analysis of particles.

Through simulation analysis, we can also realize the metastable motion analysis of particles. When the mold is completely filled, the injection molding process enters the stage of cooling and packing, and the melt gradually solidifies from the outside to the inside. At this time, there was still a certain packing effect in the cavity and the melt in the middle still had a certain shear rate and retained a low viscosity, while the melt near the cavity wall solidified more and had a higher viscosity. The particles had different resistant drag forces caused by various fluid viscosities in different regions of the cavity. In the stage of cooling and packing, a few particles can locally migrate under the action of the external magnetic field. Moreover, after the melt solidifies, the particles will stay in the sample. Subsequently, the magnetic field will not change the distribution of particles inside the sample. We regarded the motion analysis of this moment as the metastable motion analysis of particles. The metastable analysis mainly focused on the study of particles’ migration and instantaneous speed during the stage of cooling and packing, and the result is shown in Figure 11. Figure 11 depicts the metastable analysis of particles in their initial and end states, with black dots representing the particles’ initial state and cyan dots representing the particles’ end state. The particles in the legend have been magnified ten-fold their original size to make the results easier to observe. The particles were assumed to be uniformly dispersed in the cavity in order to better reflect the particles’ migration effects at various locations in the cavity. Gradients represent the different migration distances and velocities of particles during the transition from a metastable state to a steady state. The maximum distance a particle can migrate in the metastable phase is about six-fold its own size. The migration effect depicted in the diagram was supported by the theoretical analysis. During the metastable phase, particles closer to the core layer will have a superior migration distance and rate, but particles closer to the wall will migrate little, if at all. Meanwhile, the metastable analysis results show that the migration distances of particles in the cooling and packing stages are greatly reduced compared with those during the injection molding stage. The migration of particles to the near-surface of the sample mainly occurred in the injection and filling stages, which was inseparable from the lower viscosity caused by the higher shear rate during the injection molding stage. The metastable analysis results well supplemented and explained the experimental results, and the particles’ migration of the PP/Fe_3_O_4_ composite materials during the external magnetic field-assisted injection molding was divided into the injection filling stage and the cooling and packing stage, and it was found that the dominant phase of particle migration was in the injection filling stage.

## 5. Conclusions

A brand-new simulation model with multi-physics coupling was established using *COMSOL Multiphysics*^®^ simulation software, and the force and distribution of particles of functionalized PP/Fe_3_O_4_ composite materials in the fabrication of external magnetic field-assisted injection molding were investigated. All of the above findings show that magnetic particle motion is influenced by particle size, relative permeability, magnetic field intensity, and melt flow index, with particle size and magnetic field intensity having a significant impact on particle movement and being relatively easy to control during fabrication. The Carreau model was utilized to accurately incorporate melt rheological behavior from a capillary rheometer experiment into the simulation model. Though the melt flow index dose has a significant impact on particle movement, additional researches on the melt rheological behaviors of various polymers under varied forming conditions such as temperature, pressure, and cooling time are required to carry on further. The particle distribution on the experiment’s samples was approximated using the fully coupled multi-physics field simulation model, which was then compared to the experimental data and theory to ensure that the simulation results were accurate. Along with the melt injection molding process, the particle movement process is divided into the following two stages: injection filling and cooling and packing. It was discovered that particle movement occurs mostly during the injection filling stage, with an average migration distance of roughly thirty-fold greater than during the cooling and packing stage, which is primarily limited by melt cooling and solidification. The final particle distribution is only approximately 5% different from the simulation model and experimental data, which provides theoretical support for enhancing the directional migration and dispersion of magnetic microparticles in the PP-matrix magnetic composites fabrication process. The simulation model can be used to predict the motion of different particles in various polymer-matrix composite molded by injection molding assisted by the external magnetic field, as well as to improve the fabrication process to obtain more excellent surface-functionalized composites, allowing more possibilities to be explored for the application of these composite materials.

## Figures and Tables

**Table 1:** Flow properties of PP/Fe_3_O_4_ melt at 210 °C.

Parameters	Values	Units
Zero shear rate viscosity (*μ*_z_)	2800	Pa·s
Infinite shear rate viscosity (*μ*_∞_)	0	Pa·s
Relaxation time (*λ*)	0.24	s
Dimensionless parameter (*n*)	0.368	/

**Table 2:** Parameters used in the multiphysics model.

Properties	Values	Units
Magnetic Permeability of Vacuum (*μ*_0_)	4π × 10^−^^7^	N/A^2^
Recoil Permeability (*μ*_rec_)	1.05	/
Remanent Flux Density Norm (***B***_r_)	1.41	T
Density of PP (*ρ*)	900	Kg/m^3^
Melt Temperature (*T*)	483.15	K
Diameters of Particles (*d*_p_)	1	μm
Mass of Particles (*m*_p_)	8.60 × 10^−13^	g
Density of Particle (*ρ*_p_)	4800	Kg/m^3^

## Data Availability

Not applicable.

## Supplementary Materials

The following supporting information can be downloaded at: https://www.mdpi.com/article/10.3390/ma15134632/s1, Video S1: the whole processes of injection molding process with external magnetic field assisted (AVI); Video S2: the whole processes of normal injection molding process (AVI).
